# Text-Graph Enhanced Knowledge Graph Representation Learning

**DOI:** 10.3389/frai.2021.697856

**Published:** 2021-08-17

**Authors:** Linmei Hu, Mengmei Zhang, Shaohua Li, Jinghan Shi, Chuan Shi, Cheng Yang, Zhiyuan Liu

**Affiliations:** ^1^ Department Computer Science, Organization Beijing University of Posts and Telecommunications, Beijing, China; ^2^ Department High Performance Computing, Organization A*STAR, Singapore, Singapore; ^3^ Department Computer Science, Organization Tsinghua University, Beijing, China

**Keywords:** knowledge graph, graph neural networks, representation learning, graph, structure sparsity

## Abstract

Knowledge Graphs (KGs) such as Freebase and YAGO have been widely adopted in a variety of NLP tasks. Representation learning of Knowledge Graphs (KGs) aims to map entities and relationships into a continuous low-dimensional vector space. Conventional KG embedding methods (such as TransE and ConvE) utilize only KG triplets and thus suffer from structure sparsity. Some recent works address this issue by incorporating auxiliary texts of entities, typically entity descriptions. However, these methods usually focus only on local consecutive word sequences, but seldom explicitly use global word co-occurrence information in a corpus. In this paper, we propose to model the whole auxiliary text corpus with a graph and present an end-to-end text-graph enhanced KG embedding model, named Teger. Specifically, we model the auxiliary texts with a heterogeneous entity-word graph (called text-graph), which entails both local and global semantic relationships among entities and words. We then apply graph convolutional networks to learn informative entity embeddings that aggregate high-order neighborhood information. These embeddings are further integrated with the KG triplet embeddings via a gating mechanism, thus enriching the KG representations and alleviating the inherent structure sparsity. Experiments on benchmark datasets show that our method significantly outperforms several state-of-the-art methods.

## 1 Introduction

Knowledge Graphs (KGs) such as Freebase Bollacker et al. (2008) and YAGO Suchanek et al. (2007) have been widely adopted in a variety of NLP tasks. Typically, a KG consists of a set of triplets {(*h*, *r*, *t*)} where *h*, *r*, *t* stand for the head entity, relationship and tail entity, respectively.

Based on the symbolic representation of KGs with triples, a variety of methods have been designed for KG applications. As KG size increases, these methods are becoming infeasible on large-scale KGs due to computation inefficiency and data sparsity. To address the challenge, representation learning for KGs has been proposed to project the entities and relations into a continuous low-dimensional vector space. The embeddings in the latent space can significantly promote computations of the semantic distances between entities and have been proved to be helpful for knowledge graph completion, information extraction and recommender systems (Hoffmann et al., 2011; Bordes et al., 2013b; Wang X. et al., 2019).

Existing representative methods for KG embedding have achieved promising results (Lin et al., 2015; Dettmers et al., 2018). Nevertheless, these methods only exploit the structural information (i.e., existing triplets) within a KG, which is inevitably sparse and incomplete. Many entities only appear in a few triplets, making it difficult to learn good representations. To address this issue, some researches have incorporated additional information (e.g., textual descriptions) to enrich the KG representations (Socher et al., 2013; Wang et al., 2014a; Xie et al., 2016; Xu et al., 2017; An et al., 2018). For example, Xu et al. (2017); An et al. (2018) applied LSTM to encode the semantics of entity descriptions, and further learned knowledge representations with both triplets and descriptions. However, there are two limitations of these works: 1) They can only capture the local semantics in consecutive word sequences of the short entity descriptions, but may ignore global word co-occurrence information in the whole corpus. 2) CNN and LSTM models, widely used to encode auxiliary texts in these methods, are good at capturing short-range semantics, but are less effective on capturing *long-range* semantic relationships (far apart entities or words) in the texts, which has been confirmed by (Zhang et al., 2018).

**FIGURE 1:**
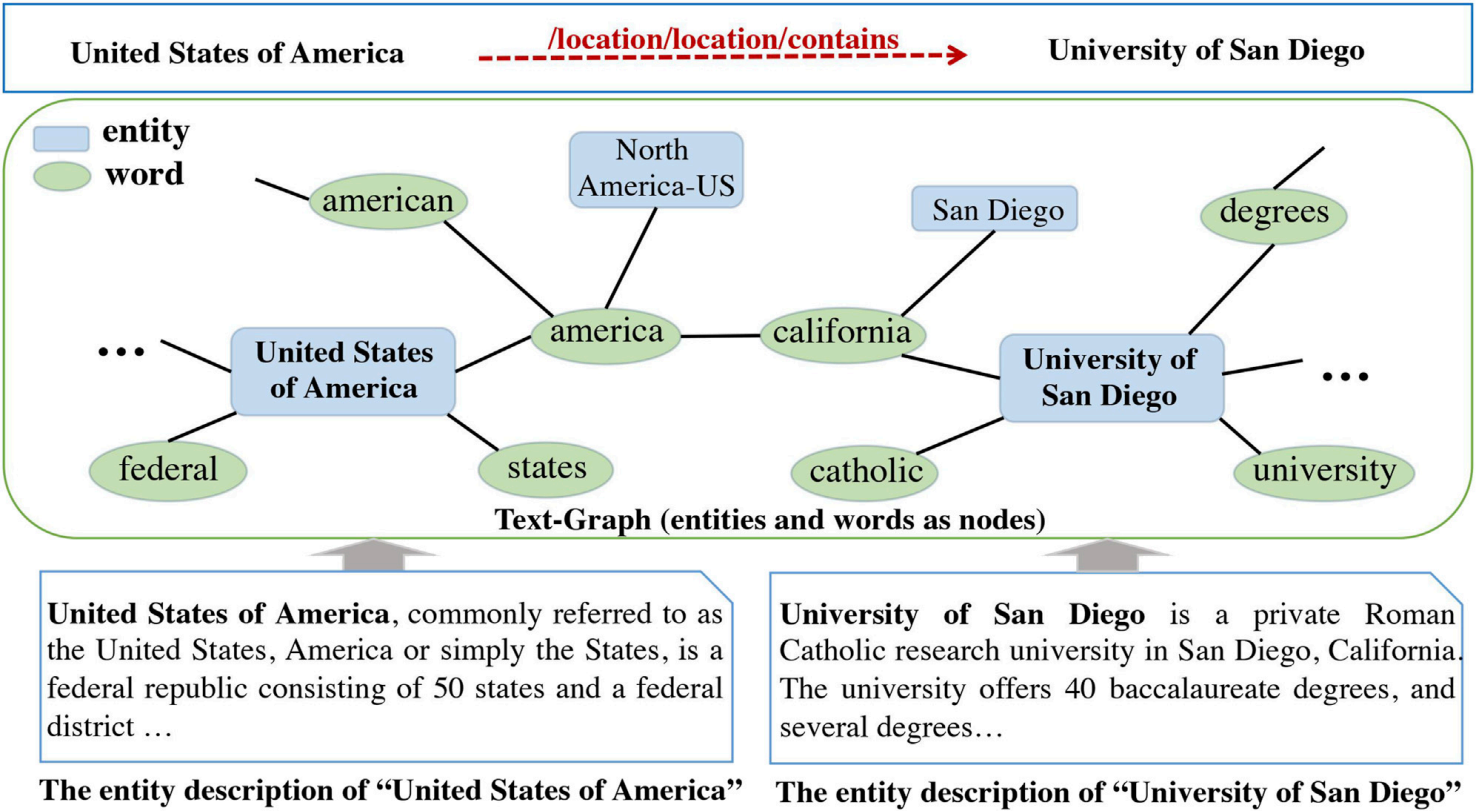
An example of text-graph. The entity University of San Diego is connected with the entity San Diego by words in their text descriptions. Leveraging the semantics in the text-graph could benefit the prediction of the link (United States of America,/location/location/contains, University of San Diego).

To address the above two limitations, we propose to model the whole auxiliary text corpus with a graph and present a novel end-to-end ***Te***xt-***g***raph ***e***nhanced KG representation learning model Teger. In particular, we model the text corpus with a heterogeneous entity-word graph (called text-graph in this paper), as shown in Figure 1. The edge between an entity and a word is built if the word occurs in the text description of the entity (local semantics). The edges between two words are built according to their global co-occurrence information in the text corpus (global semantics). In this way, far apart words in a same entity description can be bridged by the entity, which better captures the long-range relations. Based on the text-graph, Graph Convolutional Network (GCN) (Kipf and Welling, 2017), a simple and effective graph neural network which is able to capture high-order neighborhood information, is employed to encode the textual information for entity embeddings. Thus, our model is able to capture both local and global long-range semantic relationships among entities and words. Next, the entity representations learned by GCN are integrated with existing triplet embeddings using a learnable gating function and form the final entity embeddings. The whole model can be trained in an end-to-end fashion.

Figure 1 shows an example of the text-graph constructed from the auxiliary texts. The entity *“*University of San Diego” is related to the word “california” (contained in the auxiliary texts), which is then connected to a word “america” based on their similarity. Bridged by these intermediate words and entities, the relationship “location/location/contains” between the entities “University of San Diego” and *“*United States of Americ*a”* is more likely to be predicted. This example verifies the necessity of modeling global long-range relationships among entities and words.

In contrast to the aforementioned text-enhanced methods for KG embedding, our model based on a text-graph can better exploit both the local and global semantics of auxiliary texts. As a consequence, the entity representations learned by GCN based on the auxiliary text-graph can better expand the KGs while reducing the KG sparsity.

To summarize, our contributions are threefold:

1) To the best of our knowledge, we are the first to model the whole auxiliary texts with a text-graph and apply GCN for information propagation, better preserving both local and global long-range semantics of the texts.
2) We propose a novel end-to-end text-graph enhanced KG representation learning model Teger, which alleviates the structure sparsity of KGs by fully exploiting the text information represented as a text-graph.
3) After being validated on popular benchmark datasets, Teger is shown to have achieved the state-of-the-art performance, and significantly outperforms previous text-enhanced models.

The rest of this paper is organized as follows. In Section 2, we review the related work and Section 3 details our proposed model Teger. Section 4 presents the experiments and result analysis. Finally, we conclude our work in Section 5.

## 2 Related Work

This section reviews the relevant works on KG representation learning and graph neural networks.

### 2.1 KG Representation Learning

In recent years, an abundance of methods have been proposed for representation learning of KG. Translation-based models, as a powerful paradigm, have achieved promising performance in downstream tasks. TransE (Bordes et al., 2013b) regards a relationship as a translation from a head entity to a tail entity. However, it performs poorly on modeling 1-to-N, N-to-1 and N-to-N relationships. To mitigate this problem, a lot of variants of TransE have been proposed. For example, TransH (Wang et al., 2014b) regards a relationship as a hyperplane and projects head and tail entities into a relational-specific hyperplane. TransR (Lin et al., 2015) associates each relationship with a specific space when learning embeddings. TransD (Ji et al., 2015) further simplifies TransR by decomposing the projection matrix into a product of two vectors. TransG (Xiao et al., 2016) models entities as random variables with Gaussian distributions considering the uncertainty of entities. Recently, RotatE (Sun et al., 2019) extends translation-based models by representing relations as rotations in complex vector space.

Apart from translation-based models, semantic matching models using similarity-based scoring function have been explored (Yang et al., 2015; Trouillon et al., 2017; Kazemi and Poole, 2018; Bansal et al., 2019). There are also convolution based models for knowledge representation learning (Dettmers et al., 2018; Nathani et al., 2019). ConvE (Dettmers et al., 2018) applies a multi-layer convolutional network as the scoring function. GCN based models (Vashishth et al., 2020; Zhang et al., 2020) are proposed to further cover the hidden information in local neighborhood surrounding a triplet. Moreover, some recent works (Qu et al., 2021; Niu et al., 2020) design sophisticated scoring functions for reasoning on knowledge graphs, based on logic rules or reinforcement learning paradigm. One of the main limitations of the above methods is that they only utilize triplets in the KGs and suffer from the structure sparsity of the KGs.

To address the KG sparsity, text-enhanced KG representation has been extensively studied as a powerful augmentation method. For example, Socher et al. (2013) proposed a neural tensor network model exploiting the average word embeddings of an entity’s name to enhance its representation. Wang et al. (2014a) utilized entity names and Wikipedia anchors to align the embeddings of entities and words in the same space. Malaviya et al. (2020) exploited BERT to encode the entity names of commonsense KG. Zhong et al. (2015); Zhang et al. (2015); Veira et al. (2019) improved the model of (Wang et al., 2014a) with a new alignment model based on entity descriptions without utilizing anchors. However, these methods struggle with ambiguity within entity names. Hence Xie et al. (2016); Wang Z. et al. (2019) learned knowledge representations using concise descriptions of entities instead of entity names. Xu et al. (2017) proposed a gating mechanism to integrate both structure and textual representations. An et al. (2018) leveraged both entity descriptions and relationships mentions (Riedel et al., 2013; Toutanova et al., 2015) to further improve KG embedding. Qin et al. (2020) utilized generative adversarial networks to generate KG embeddings for unseen relations merely with noisy descriptions as input. Although these methods achieve improved performance, they fail to fully exploit the semantics of auxiliary texts. In these methods, each entity can only exploit the semantic information in the local consecutive word sequence of the short description, and ignore global relationships among entities and words. Moreover, the majority of them use CNN or LSTM-based for encoding texts, which are good at modeling semantic composition but less advantageous on capturing long-range correlations between entities and words within the descriptions.

Different from the existing works, in this work, we propose to model the whole auxiliary texts of entities as a text-graph and present a novel end-to-end text-graph enhanced KG representation learning model.

### 2.2 Graph Neural Networks

Graph Neural Networks have received wide attention recently. GCN (Kipf and Welling, 2017) has shown its power in embedding graph structures by enabling information propagating from neighboring nodes. The recent works utilize GCNs to encode more complicated pairwise relationships between entity/tokens. It has been proven that there is a rich variety of NLP problems that can be best expressed with a graph structure (Wu et al., 2021). Yao et al. (2019) proposed a GCN-based model viewed documents and words as nodes of a graph, allowing word and document embeddings jointly learned. Zhang et al. (2018) improved the performance of relationship extraction by utilizing GCN over dependency trees. Bastings et al. (2017) employed GCN to encode syntactic structure of sentences for machine translation. Some recent studies (Schlichtkrull et al., 2018) start to explore graph neural networks for knowledge base completion task, considering only the structural information of the KGs.

In this paper, we model the texts of entities as a graph and apply GCN for obtaining informative entity embeddings that encode textual information, in order to expand the KGs and alleviate the structure sparsity.

## 3 The Proposed Method

This section depicts our proposed text-graph enhanced KG representation learning model Teger. Teger improves tradional KG embeddings (e.g., TransE) by fully exploiting the auxiliary texts of entities (e.g., entity descriptions) which are represented as a text-graph, capturing both local and global long-range semantics of the texts.

**FIGURE 2:**
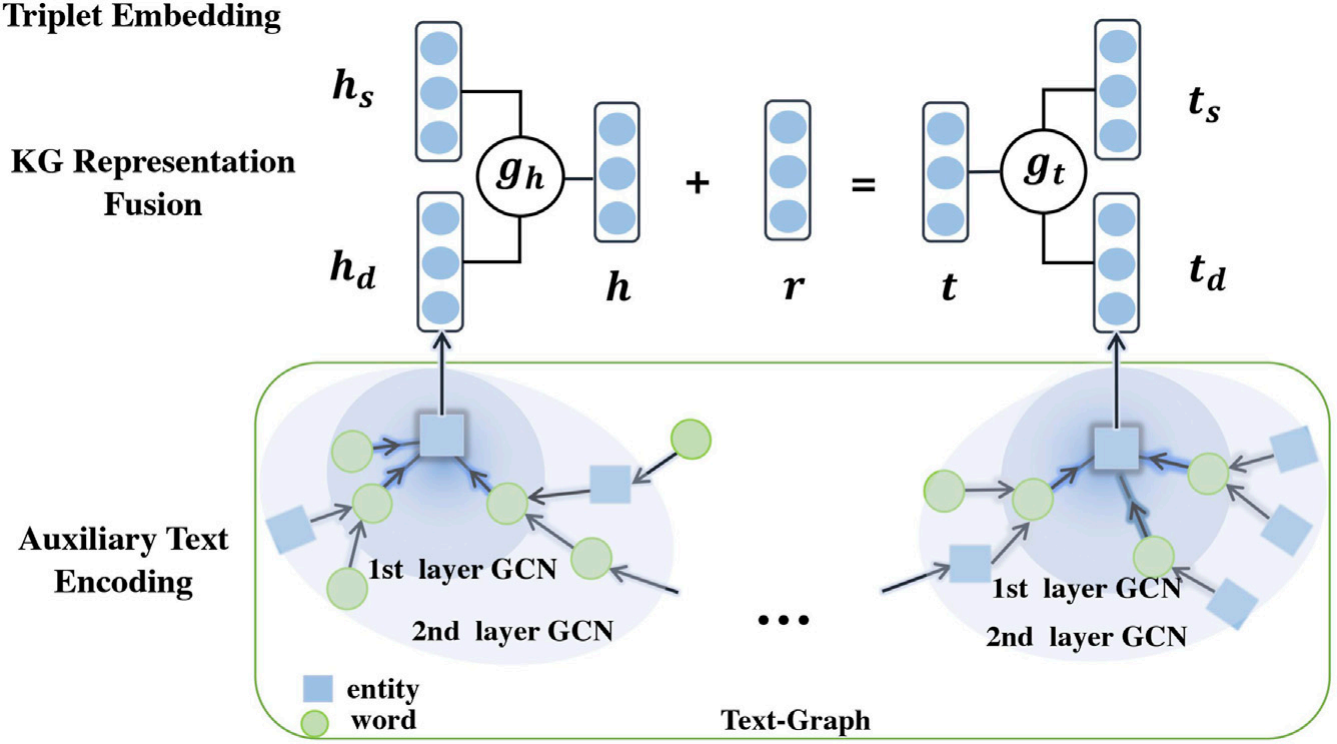
Illustration of Teger for text-graph enhanced KG embedding.

Specifically, Teger consists of three components: (1) Triplet embedding. The triplet embedding aims to obtain structural entity embeddings (We use TransE as an exmaple in this work). (2) Auxiliary text encoding, which is to encode the semantics from auxiliary texts to enrich the KG. To capture both the local and global semantic relationships among entities and words, we first construct a text-graph from the auxiliary texts and then apply GCN to get entity embeddings by aggregating neighboring semantic information. (3) KG representation fusion. The GCN-yielded embeddings are furhter integrated with triplet embeddings through a gating mechanism, which alleviates the structure sparsity of the KGs. Figure 2 illustrates the three components of Teger.

### 3.1 Triplet Embedding

Teger is a general framework to enhance existing triplet embedding methods. In this paper, we take TransE (Bordes et al., 2013b) as an example.

Formally, given a triplet (*h*, *r*, *t*), TransE maps entities *h*, *t* and the relationship *r* to embedding vectors **h**, **t**, **r** in the same space, and requires that the embedding **t** to be close to **h** + **r** if (*h*, *r*, *t*) holds. The score function of TransE is defined as the distance between **h** + **r** and **t**:

$$f\left(h,r,t\right)=-\|h+r-t{\|}_{2}^{2},$$ (1)

where **h** and **t** are subject to the normalization constraint that the magnitude of each vector is 1. In this form, relationships are represented as translations in the embedding space: if (*h*, *r*, *t*) holds, the embedding of the tail entity *t* should be close to the embedding of the head entity *h* plus relationship vector *r*.

### 3.2 Auxiliary Text Encoding

This section presents our proposed auxiliary text encoding scheme. We first detail how a text-graph is constructed from the auxiliary texts of entities in a given KG, and then present the graph convolutional encoder for obtaining entity embeddings that encode the textual information.

Text-Graph Construction. To better exploit global and long-range semantic relationships in the auxiliary texts, we build a heterogeneous entity-word graph (called text-graph) from the texts, *G* = {*V*, *E*} where *V* represents the nodes including entities E and words W, and *E* denotes the edges. As shown in Figure 1, the text-graph ecodes both local and global long-range semantic relationships among entities and words.

Specifically, for each entity e∈E, we first select *K* words *w*_1_, …, *w*_*K*_ with the highest TF-IDF values in the description of *e* as word nodes, and build edges between the entity *e* and *w*_1_, …, *w*_*K*_. To incorporate global semantics among entities and words, we further build edges between word pairs if their similarity score is above a predefined threshold *δ*. In this work, we compute the similarity score between a word pair based on the pre-trained word embeddings using Word2Vec on Google News dataset^1^. Figure 1 is an example of entity-word graph *G* = {*V*, *E*} constructed from the auxiliary texts.

Graph Convolutional Encoder. After constructing the text-graph from the auxiliary texts, GCN which is effective in capturing high-order neighborhood information, is applied to learn the representations of entities that aggregate high-order semantic information. Note that we apply TransE to obtain pre-trained entity embeddings **e**, and then initialize the embedding **w** of a word by averaging the its 1-hop neighboring entity embeddings in the graph *G*. In this way, the input embeddings of entities and words are in the same semantic space, thus we can directly apply GCN on the text-graph.

Formally, consider the text-graph *G* = {*V*, *E*} where *V* and *E* represent the set of nodes (including entities and words) and edges respectively. We introduce an adjacency matrix *A* of *G* and its degree matrix *D*, where *D*_*ii*_ = Σ_*j*_ *A*_*ij*_, where the diagonal elements of *A* are set to 1 with self-loops. Let $X\in R^{M\times N}$ be the matrix containing the pre-trained embeddings of all the nodes (each row is a feature vector **x**_*v*_ for a node *v*), the embeddings *H* ^(*l*+1)^ of all the nodes are updated as follows:

$$H^{\left(l+1\right)}=\sigma \left(D^{-\frac{1}{2}}\tilde{A}D^{-\frac{1}{2}}H^{\left(l\right)}W^{\left(l\right)}+H^{\left(l\right)}\right),$$ (2)

where *H* ^(*l*)^ is the hidden states of nodes in the *l* ^*th*^ layer, *σ*(⋅) is a non-linear activation function. Initially, *H* ^(0)^ is set to *X*. Intuitively, multiplication with $D^{-\frac{1}{2}}\tilde{A}D^{-\frac{1}{2}}$ means that, for every node, we smooth its feature with all the feature vectors along graph structure. The addition of *H* ^(*l*)^ represents a simple skip-connection, further encouraging preservation of information about the central node.

After going through an *L*-layer GCN, we get a new set of entity embeddings which aggregate semantics from their neighbors in the text-graph. The entity embeddings encode both local and global semantics from the auxiliary texts, which will enrich the KG and alleviate its structure sparsity.

### 3.3 KG Representation Fusion

In this section, we describe how to obtain final KG embeddings that combine both the textual information of the auxiliary texts and the structural information of triplets in the KG.

Specifically, as we have *e*_*d*_ encoding the auxiliary texts and *e*_*s*_ (based on TransE/ConvE) encoding the structural information (i.e., triplets) in the KG, we adopt a learnable gating function (Xu et al., 2017) to integrate entity embeddings from the two sources. Formally,

$$e=g_{e}⊙e_{s}+\left(1-g_{e}\right)⊙e_{d},$$ (3)

where **g**_*e*_ is a gating vector to trade-off information from two sources with all elements in [0, 1], and ⊙ is element-wise multiplication. We assign a gate vector **g**_*e*_ to each entity *e*, which means each dimension of **e**_*s*_ and **e**_*d*_ for entity *e* are summed by different weights. To constrain that the value of each element is in [0, 1], we compute the gate with the sigmoid function:

$$g_{e}=\sigma \left({\tilde{g}}_{e}\right),$$ (4)

where ${\tilde{g}}_{e}$ is a real-value vector and is learned in the training process.

After fusing the two types of embeddings with the gating function, we obtain the final entity embeddings which encode both textual information from the auxiliary texts and structural information from triplets in the KG. Compared to existing triplet embedding methods, Teger expands the KG by exploiting both local and global semantic relationships extracted from the auxiliary texts, with the aim to alleviate the KG sparsity problem.

### 3.4 End-to-End Model Training

We train the model parameters, including the weight matrices of GCN, the gating vectors and the word, entity and relation embeddings in an end-to-end fashion by minimizing the following loss function *L*:

$$\begin{array}{cc} L=\underset{\left(h,r,t\right)\in S}{\Sigma }\underset{\left(h^{\prime },r,t^{\prime }\right)\in S^{\prime }}{\Sigma }ma & x\left(\right.\gamma +f\left(h,r,t\right)\\ - & f\left(h^{\prime },r,t^{\prime }\right),0\left.\right), \end{array}$$ (5)

where *S* is the collections of correct triplets, *S*′ is the set of incorrect triplets, and *γ* is the margin between correct and incorrect triplets. The triplet set *S*′ is the negative sampling set of *S* by replacing the head or tail entity in correct triplets. We follow the sampling strategy “bern” in (Wang et al., 2014b) to generate negative samples. Such a margin-based ranking loss can encourage discrimination between golden triplets and incorrect triplets. The scoring function *f* (*h*, *r*, *t*) for a triplet (*h*, *r*, *t*) is defined as:

$$\begin{array}{cc} f\left(h,r,t\right) & =\|\left(g_{h}⊙h_{s}+\left(1-g_{h}\right)⊙h_{d}\right)+\\ & r-\left(g_{t}⊙t_{s}+\left(1-g_{t}\right)⊙t_{d}\right){\|}_{2}^{2}, \end{array}$$ (6)

where **g**_**h**_ and **g**_**t**_ are the gating vectors of entity *h* and *t* respectively. We use Adam (Kingma and Ba, 2014) for model optimization.

## 4 Experiments

In this section, we evaluated the performance of our proposed method Teger on the tasks of link prediction and triplet classification, against state-of-the-art baseline methods.

### 4.1 Experimental Setup

Datasets. We evaluated our method Teger on two knowledge bases: FB15K which is a subset of Freebase Bollacker et al. (2008) and WN18 (Bordes et al., 2013b) which is a subset of WordNet. Both datasets come with textual descriptions of each entity, which we use as the auxiliary texts. Specifically, WordNet is a large lexical database of English with each entity as a synset which consists of several words and corresponds to a distinct word sense. Freebase is a large knowledge graph of general world facts. The dataset FB15K^2^ is offered by (Xie et al., 2016), which extracts a short description for each entity from its corresponding wiki-page. In FB15K, the average length of the entity descriptions is 69 after removing stop words. While for the dataset WN18, the length of entity descriptions is smaller, containing 13 words in average. The statistics of the datasets are detailed in Table 1. Note that for KGs where entity descriptions are absent, one can take the entities as queries and extract short text snippets describing the queries with search engines.

**TABLE 1:** Statistics of the datasets.

Dataset	#Relationship	#Entity	#Word	#Train	#Valid	#Test
FB15K	1,341	14,904	28,383	472,860	48,991	57,803
WN18	18	40,493	30,519	141,442	5,000	5,000

Baselines. We compared Teger with the state-of-the-art KG embedding methods as follows:

• Basic Models: We compared Teger with some basic models, learning KG representation without text information. Among which, TransE (Bordes et al., 2013b) is a classic translation-based knowledge graph embedding model. UnS (Unstructured model) (Bordes et al., 2012) is a simplified version of TransE by setting all **r** = **0**. TransH (Wang et al., 2014b) improves TransE by introducing relationship-specific hyperplanes. TransR (Lin et al., 2015) introduces relationship-specific spaces. TransD (Ji et al., 2015) further simplifies TransR by decomposing the projection matrix into a product of two vectors. SME (Bordes et al., 2013a) uses similarity-based scoring functions with neural network architectures. There are two versions of SME: a linear version SME (linear) and a bilinear version SME (bilinear). We also generalize our model Teger to the state-of-the-art method ConvE (Dettmers et al., 2018) which employs a convolutional network model as scoring function, and compare it (i.e., Teger_ConvE) with ConvE.
• Text-Enhanced Models: Text-enhanced models incorporate textual information for KG representation learning. We compared our model Teger with state-of-the-art text-enhanced models based on TransE including J (LSTM)/J (A-LSTM) Xu et al. (2017) and AATE_E (An et al., 2018). J (LSTM)/J (A-LSTM) uses LSTM or attention-based LSTM to encode the descriptions. AATE_E uses mutual attention based LSTM to learn textual embeddings from both entity descriptions and English Wikipedia pages.

Parameter Settings. We selected the threshold *δ* among {0.2, 0.4, 0.6, 0.8}, the top *K* words for each entity among {5, 10, 15, 20}, the margin *γ* among {1, 2, 4}, the embedding dimension *d* among {20, 40, 100}, the learning rate *λ* among {0.0001, 0.001, 0.01, 0.1}, batch size *b* among {1,000, 3,000, 5,000, 10,000}, the layers of GCN *L* among {1, 2, 3}. The activation function *σ*(⋅) in GCN is set to *tanh* (⋅). The best configurations obtained through experiments on validation set are shown in Table 2.

**TABLE 2:** Parameter settings.

Parameter	Value	
	FB15K	WN18
Top *K* words	5	5
Threshold *δ*	0.6	0.6
Margin *γ*	4	4
Embedding dimension *d*	100	40
Learning rates *λ*	0.0001	0.0001
Batch size *b*	10,000	3,000
Layers of GCN *L*	2	2

### 4.2 Link Prediction

Link prediction is a subtask of knowledge graph completion, which aims to predict missing *h* or *t* in a triplet (*h*, *r*, *t*). For each missing entity, this task is to give a ranked list of candidate entities from the KG, rather than just guessing the best answer. Following (Bordes et al., 2013b), we conducted experiments on FB15K and WN18.

Since there are only correct triplets in the KG, we constructed corrupted triplets (*h*′, *r*, *t*′) in KGs for a triplet (*h*, *r*, *t*) by randomly replacing the head/tail entity with other entities using Bernoulli Sampling (Wang et al., 2014b). Then we ranked these entities in descending order by the scoring function *f*. Given the entity ranking list, we employed two evaluation metrics (Bordes et al., 2013b): (1) the average rank of correct entities (MR); (2) the proportion of correct entities in the top-10 ranked entities Hits@10. Corrupted triplets may also exist in the KG, and such a prediction should not be regarded as an error. Thus, following (Bordes et al., 2013b), we removed those corrupted triplets which appear in either training, validation or test sets before getting the ranking lists. The overall results are presented in Table 3.

**TABLE 3:** Results on link prediction. MR is the lower the better; Hits@10 is the higher the better.

Models	WN18		FB15K	
	MR *↓*	Hits10 *↑*	MR *↓*	Hits10 *↑*
UnS	304	38.2	979	6.3
SME (linear)	533	74.1	154	40.8
SME (bilinear)	509	61.3	158	41.3
TransH	303	86.7	84	58.5
TransR	225	92.0	77	68.7
TransD	212	92.2	91	77.3
TransE	251	89.2	125	47.1
AATE_E	123	94.1	76	76.1
J (LSTM)	95	91.6	90	69.7
J (A-LSTM)	123	90.9	73	75.5
Teger_TransE	168	94.7	72	76.3
ConvE	374	95.6	51	83.1
Teger_ConvE	336	95.6	47	85.1

Overall Results. In Table 3, one can observe that our model Teger_TransE significantly outperforms TransE, which indicates that knowledge representation can greatly benefit from text descriptions. Furthermore, Teger_TransE achieves better performance than other text-enhanced methods based on TransE, without an attention mechanism. The improvements of Teger_TransE over J (LSTM) and J (A-LSTM) models which use the same entity descriptions demonstrate that Teger better exploits the semantics of auxiliary texts. Teger benefits from the text-graph constructed from auxiliary texts, which captures the global relationships among entities and words. Teger_TransE also outperforms AATE_E, which use long Wikipedia articles corresponding to the entities (Shaoul, 2010) (containing 495 words on average). This further demonstrates the effectiveness of Teger in making full use of limited text information. In future work, we could explore to utilize such longer textual information.

We can also see from Table 3 that our model Teger_TransE achieved comparable performance to the state-of-the-art models including TransD and ConvE. It is worth noting that our proposed framework Teger can be transferred to these models, further improving their performance. We explored the transfer of Teger to the best baseline model ConvE and found that Teger_ConvE achieves better results than ConvE on both datasets. It further demonstrates the effectiveness of our text-graph enhanced KG embedding model. Note that Teger is based on real-vector space, which cannot be directly generalized to complex-vector based models, such as RotatE (Sun et al., 2019). We would like to extend our model to complex vector space in the future.

On the WN18 dataset, we observed that the mean rank (MR) of Teger_TransE is worse than the state-of-art models. The reason may be that MR could be largely influenced by one extremely bad case, while the metric of Hits@10 would not. Since the entity descriptions of some entities in the data set are quite short, e.g., containing only one word, the semantic propagation may be limited.

Detailed Results on Different Types of Relationships. To further analyze the effect of our model Teger_TransE, following Bordes et al. (2013b) and (Han et al., 2018), we divided the relationships into four types: 1-to-1, 1-to-N, N-to-1 and N-to-N, for which the proportions in FB15K are 26.3*%*, 22.7*%*, 28.2*%* and 22.8*%* respectively. Table 4 presents the results of Teger_TransE on four types of relationships on link prediction task.

**TABLE 4:** Results on FB15K by the category of relationships.

Tasks	Prediction Head (Hits@10)				Prediction Tail (Hits@10)			
**Relationship Category**	**1-to-1**	**1-to-N**	**N-to-1**	**N-to-N**	**1-to-1**	**1-to-N**	**N-to-1**	**N-to-N**
TransE	43.7	65.7	18.2	47.2	43.7	19.7	66.7	50.0
TransH	66.8	87.6	28.7	64.5	65.5	39.8	83.3	67.2
TransD	86.1	95.5	39.8	78.5	85.4	50.6	94.4	81.2
AATE_E	-	96.1	35.2	49.1	-	32.2	98.3	60.3
J (LSTM)	81.3	88.9	18.8	45.2	80.1	25.4	89.6	52.4
J (A-LSTM)	83.8	95.1	21.1	47.9	83.0	30.8	94.7	53.1
Teger_TransE	87.3	96.3	54.1	75.9	84.9	54.9	95.5	79.1

Experimental results in Table 4 show that our model Teger_TransE achieve the best perfomance on most cases. All the models including AATE_E, J (LSTM), J (A-LSTM) which extend TransE to incorporate auxiliary texts consistently outperform TransE. It demonstrates that the textual information can effectively enrich the semantics of a KG, alleviating its structure sparsity and learning better KG embeddings. It is worth noting that compared to the baseline method TransE, our model Teger_TransE achieves significant performance gains. It shows that by making full use of the semantic relationships within short text descriptions through GCN, we are able to learn KG representations of much higher quality. It can also be observed that Teger_TransE outperforms all the other TransE based text-enhanced models on almost all the categories of relationships. We believe the reason is that Teger better exploits the semantic information from auxiliary texts by modeling the texts as a graph which captures both local and global long-range semantic relationships among entities and words.

### 4.3 Triplet Classification

In this section, we evaluated different methods on the triplet classification task, which aims to confirm whether a given triplet (*h*, *r*, *t*) is correct or not. Following Socher et al. (2013) and (Han et al., 2018), we created negative triplets by replacing entities. For the classification of a triplet (*h*, *r*, *t*), we classified it as “correct” when the score of the triplet is equal or greater than a predefined threshold *T*_*r*_. The threshold *T*_*r*_ for a relationship *r* is determined by maximizing the classification accuracy on the validation set.

Table 5 presents the results of triplet classification on FB15K and WN18. As we can see, on FB15K, all the text-enhanced methods outperform the triplet embedding methods based on only structure information. In addition, all the TransE based text-enhanced models including J (LSTM), J (A-LSTM) and our Teger_TransE significatly outperform TransE on both datasets. These observations demonstrate the effectiveness of leveraging auxiliary texts to enrich KG embeddings. Our model Teger_TransE obtains the best performance on WN18, while achieves inferior performance on FB15K. The reason could be that the entity descriptions in WN18 are shorter, which can benefit more from global semantic information.

**TABLE 5:** Results on triplet classification.

Datasets	WN18	FB15K
TransE	92.9	79.8
TransH	−	79.9
TransR	−	82.1
TransD	−	88.0
J (LSTM)	97.7	90.5
J (A-LSTM)	97.8	91.5
Teger_TransE	98.1	89.5

## 5 Conclusion and Future Work

In this paper, we propose Teger, a novel end-to-end text-graph enhanced knowledge graph representation method. Teger enriches the KG embedding by effectively incorporating the auxiliary text information represented by a graph. Particularly, we first construct a text-graph from the auxiliary text and then apply GCN to obtain entity embeddings by aggregating neighboring information, which can capture both local and global semantic relationships among entities and words. The GCN-yielded embeddings are then integrated with a gating mechanism to augment existing KG embeddings based on triplets and alleviate the structure sparsity of the KG. Experiments on two benchmark datasets demonstrate the superiority of Teger for text-enhanced KG embedding by representing the auxiliary texts as a graph and effectively incorporating the textual information.

In future work, we will explore to apply graph attention networks for encoding the text-graph, considering that there could be some noise in the texts. It would also be interesting to generalize Teger to complex-vector space.

## Data Availability

^3^The original contributions presented in the study are included in the article/supplementary material, further inquiries can be directed to the corresponding author.

## Footnotes

1
https://drive.google.com/file/d/0B7XkCwpI5KDYNlNUTTlSS21pQmM/

2
https://github.com/xrb92/DKRL
